# Clinical and laboratory-induced colistin-resistance mechanisms in *Acinetobacter baumannii*

**DOI:** 10.1099/mgen.0.000246

**Published:** 2019-02-05

**Authors:** Christine J. Boinett, Amy K. Cain, Jane Hawkey, Nhu Tran Do Hoang, Nhu Nguyen Thi Khanh, Duy Pham Thanh, Janina Dordel, James I. Campbell, Nguyen Phu Huong Lan, Matthew Mayho, Gemma C. Langridge, James Hadfield, Nguyen Van Vinh Chau, Guy E. Thwaites, Julian Parkhill, Nicholas R. Thomson, Kathryn E. Holt, Stephen Baker

**Affiliations:** ^1^​ Wellcome Trust Sanger Institute, Wellcome Trust Genome Campus, Hinxton, Cambridge CB10 1SA, UK; ^2^​ Hospital for Tropical Diseases, Wellcome Trust Major Overseas Programme, Oxford University Clinical Research Unit, Ho Chi Minh City, Vietnam; ^3^​ Centre for Tropical Medicine and Global Health, Nuffield Department of Clinical Medicine, Oxford University, Oxford, UK; ^4^​ Malawi-Liverpool-Wellcome Trust Clinical Research Programme, University of Malawi College of Medicine, Blantyre, Malawi; ^5^​ Centre for Systems Genomics, University of Melbourne, Melbourne, Victoria, Australia; ^6^​ Department of Biochemistry and Molecular Biology, Bio21 Molecular Science and Biotechnology Institute, University of Melbourne, Melbourne, Victoria, Australia; ^7^​ Faculty of Veterinary and Agricultural Sciences, University of Melbourne, Melbourne, Victoria, Australia; ^8^​ School of Chemistry and Molecular Biosciences, University of Queensland, Brisbane, Queensland, Australia; ^9^​ Department of Biology, Drexel University, Philadelphia 19104, PA, USA; ^10^​ Hospital for Tropical Diseases, Ho Chi Minh City, Vietnam; ^11^​ Norwich Medical School, University of East Anglia, Norwich, UK; ^12^​ Department of Infectious and Tropical Diseases, London School of Hygiene and Tropical Medicine, London, UK; ^13^​ Medicine, The University of Cambridge, Cambridge, UK

**Keywords:** *Acinetobacter baumannii*, colistin resistance, multi-drug resistance, RNAseq, TraDIS, whole-genome sequencing

## Abstract

The increasing incidence and emergence of multi-drug resistant (MDR) *Acinetobacter baumannii* has become a major global health concern. Colistin is a historic antimicrobial that has become commonly used as a treatment for MDR *A. baumannii* infections. The increase in colistin usage has been mirrored by an increase in colistin resistance. We aimed to identify the mechanisms associated with colistin resistance in *A. baumannii* using multiple high-throughput-sequencing technologies, including transposon-directed insertion site sequencing (TraDIS), RNA sequencing (RNAseq) and whole-genome sequencing (WGS) to investigate the genotypic changes of colistin resistance in *A. baumannii.* Using TraDIS, we found that genes involved in drug efflux (*adeIJK*), and phospholipid (*mlaC, mlaF* and *mlaD*) and lipooligosaccharide synthesis (*lpxC* and *lpsO*) were required for survival in sub-inhibitory concentrations of colistin. Transcriptomic (RNAseq) analysis revealed that expression of genes encoding efflux proteins (*adeI*, *adeC*, *emrB*, *mexB* and *macAB*) was enhanced in *in vitro* generated colistin-resistant strains. WGS of these organisms identified disruptions in genes involved in lipid A (*lpxC*) and phospholipid synthesis (*mlaA*), and in the *baeS*/*R* two-component system (TCS). We additionally found that mutations in the *pmrB* TCS genes were the primary colistin-resistance-associated mechanisms in three Vietnamese clinical colistin-resistant *A. baumannii* strains. Our results outline the entire range of mechanisms employed in *A. baumannii* for resistance against colistin, including drug extrusion and the loss of lipid A moieties by gene disruption or modification.

## Data Summary

1. The PacBio (Pacific Biosciences) sequence data and assembly for BAL062 can be found at the European Nucleotide Archive (ENA) under accession numbers: ERR581111 andERR581112, ERR581112 (www.ebi.ac.uk/ena).

2. Genome BAL062 has been deposited in GenBank under accession numbers LT594095 (chromosome) and LT594096 (plasmid) (url: www.ebi.ac.uk/ena/data/view/LT594095 and www.ebi.ac.uk/ena/data/view/LT594096).

3. RNA sequencing, transposon-directed insertion site sequencing and whole-genome sequencing data is available at the ENA (www.ebi.ac.uk/ena) under the study accession numbers detailed in Table S1 (available with the online version of this article).

**Impact Statement**

Colistin was first introduced into the clinical field in the late 1950s, but its use later declined due to toxicity. In recent years, colistin has been reintroduced as a last-line therapy in treating multi-drug resistant (MDR) Gram-negative infections, including *Acinetobacter baumannii*. The rise in colistin-resistant *A. baumannii* clinical strains has been reported and the lack of new antimicrobials in the pipeline to treat Gram-negative bacteria greatly decreases the chance of a positive outcome in treating MDR *A. baumannii* infections. We used multiple sequence-based approaches to identify the mechanisms behind the development of colistin resistance in *A. baumannii*. In addition to other known mechanisms of colistin resistance, we observed recombination events in clinical colistin-resistant *A. baumannii* strains, a novel mechanism that may contribute to fitness recovery. We also highlight the use of a high-throughput mutagenesis approach that simultaneously assays the genome for novel candidate genes involved in colistin resistance.

## Introduction

The incidence of healthcare-acquired infections caused by multi-drug resistant (MDR) and pan-drug resistant *Acinetobacter baumannii* has increased dramatically in recent years [1]. With limited alternative treatment strategies available, there has been an increasing use of the polymyxin antimicrobial, colistin, an older generation last-line antimicrobial that is frequently used alone or in combination with tigecycline, carbapenems or rifampicin [2–4]. Despite the use of combination therapy, the incidence of heteroresistance and complete resistance to colistin (col^R^) alone has been frequently reported in clinical isolates of *A. baumannii*, and can result in treatment failure [5, 6].

*In vivo* and *in vitro* studies of *A. baumannii* have identified two main genetic mechanisms for the induction of col^R^: (i) lipooligosaccharide (LOS) modification through the acquisition of single-nucleotide polymorphisms (SNPs) in *pmrAB*; or (ii) the complete loss of the LOS owing to SNPs in genes encoding lipid A biosynthesis genes *lpxA, lpxC* and *lpxD* [7]. Alteration or loss of the LOS results in the reduction of the net negative charge of the LOS; thus, decreasing the affinity between colistin and the cell membrane [8–11]. Insertion sequence (IS) elements, such as ISAba1 and ISAba11, have also been associated with the development of col^R^ via the disruption of genes in the *lpx* gene cluster [12, 13].

Here, we aimed to gain insight into the genetic mechanisms associated with col^R^ in *A. baumannii* isolates from Vietnam. This type of study is essential for cataloguing the various mechanisms associated with the development of antimicrobial resistance in *A. baumannii* in clinical and *in vitro* generated col^R^ mechanisms. This is particularly relevant given the different forms of colistin used clinically, such as colistin methanosulphate for therapy and colistin sulphate for selective decontamination of the gastrointestinal tract [14]. Previous studies have utilized genomic and transcriptomic analysis of *in vivo* and *in vitro* induced col^R^ mutants to determine mechanisms associated with resistance; however, genome-wide high-throughput mutagenesis has not been conducted. In this study, we used a colistin susceptible (col^S^) *A. baumannii* strain (BAL062) to generate a mutant library to assay for genes required for survival in sub-inhibitory concentrations of colistin. Additionally, we used a controlled directed-evolution approach to generate a col^R^ variant from a colistin-susceptible MDR *A. baumannii* isolated from a patient with ventilator-associated pneumonia (VAP) on an adult intensive care unit in a Vietnamese hospital to investigate the genetic and transcriptional changes in the col^R^ cultures [15]. We additionally performed whole-genome sequencing (WGS) on three clinical VAP col^R^ *A. baumannii* isolated between 2012 and 2013 from the same ward to assess the mechanisms and relation, if any, to *in vitro*-derived col^R^ *A. baumannii*.

## Methods

All four MDR organisms used in this study were collected as part of a larger study investigating the aetiology of VAP at the Hospital for Tropical Diseases in Ho Chi Minh City, Vietnam, in 2009 [16]. These organisms, BAL062, BAL505, BAL546 and BAL719, were isolates from bronchoalveolar lavage (BAL). BAL062 (col^S^) [17] was used to generate the transposon library and also to generate a col^R^ variant for the RNA sequencing (RNAseq) experiments. BAL505, BAL546 and BAL719 were all determined to be clinically col^R^ by disc diffusion [16] and were selected for WGS.

### Susceptibility testing

Minimum inhibitory concentrations (MICs) were determined by the broth dilution method according to methods described elsewhere [18]. MICs were interpreted according to Clinical and Laboratory Standards Institute guidelines [19].

### Inducing colistin resistance

Duplicate cultures (C1 and C2) were subjected to serial daily passage on Mueller–Hinton (MH) agar (Oxoid) plates with increasing concentrations (double dilutions) of colistin (1–128 mg l^−1^; Sigma-Aldrich) until the cultures were able to grow in 128 mg colistin l^−1^ (MIC >128 mg l^−1^), i.e. ‘endpoint’. The endpoints for C1 and C2 were day 6 and day 5, respectively. Briefly, approximately 10^9^ c.f.u. were resuspended in 100 µl PBS (0.9 %) and 3 µl was spotted onto agar plates with increasing concentrations of colistin sulphate ranging from 1 to 128 mg l^−1^ (serial dilutions) and incubated overnight at 37 °C. Colonies (>20 colonies) were taken from the plate with the highest concentration of colistin sulphate from each culture (C1/C2) and resuspended in 100 µl 0.9 % PBS, which was used to inoculate a fresh batch of plates containing colistin. This procedure was repeated for the duration of the experiment (5/6 days) until the defined endpoint. At key time points, day 0 (WT), at 32 mg colistin l^−1^, midpoint (64 mg colistin l^−1^) and endpoint (128 mg colistin l^−1^), 10 µl cell suspension was used to inoculate 10 ml fresh MH broth (Oxoid) and cultures were incubated at 37 °C with agitation. RNA and DNA were extracted at mid-log phase and after overnight incubation for RNAseq and WGS, respectively. No 32 mg colistin l^−1^ time point was collected for C2, as it achieved midpoint col^R^ after one serial passage.

### PacBio (Pacific Biosciences) sequencing of BAL062

Genomic DNA was sequenced using the PacBio RSII sequencer (PacBio), as previously described [20]. This yielded >65 094 individual reads with an *N*_50_ of 8.8 kb (post-filtering), generating at least 100× coverage. *De novo* assembly of the resulting reads was performed using HGAP.3 (PacBio). The genomes were annotated with Prokka [21] and set to start at *dnaA*. The sequence data and assembly can be found in the European Nucleotide Archive (ENA) under accession numbers ERR581111 and ERR581112, and (chromosome) LT594095 and (plasmid) LT594096.

### WGS

DNA was sequenced on an Illumina MiSeq or HiSeq2000 (Illumina) using a method described elsewhere [22]. These data have been deposited in the ENA (Table S1). The clinical isolates were mapped onto the *A. baumannii* GC2 (global clone 2) 1652–2 reference genome (GenBank accession no. CP001921.1), as with previous VAP and carriage isolates [15], to identify SNPs using a previously described method [23]. Accession numbers for the VAP and carriage isolates are shown in Table S2. SNPs in the *pmr* locus were confirmed by Sanger sequencing (Table 3) using the primers detailed in Table S3. Reads from the *in vitro* col^R^-generated organism were mapped to a complete genome sequence of BAL062. SNPs were determined as previously described using the RedDog mapping pipeline (https://github.com/katholt/RedDog) [15]. To identify regions disrupted by IS elements, the BAL062 reference genome was annotated using ISSaga (www-is.biotoul.fr/) to identify ISs for screening with ISMapper [24].

### Transcriptomics and differential expression analysis

RNA was extracted from the initial, midpoint and endpoint C1 and C2 cultures at OD_600_ 0.5+/−0.05 using a modified phenol/chloroform extraction protocol [25]. Ribosomal RNA was depleted using a Ribo-Zero Magnetic kit (Epicentre Biotechnologies). The libraries were prepared using the TruSeq Illumina protocol and sequenced on an Illumina HiSeq2000 platform. Reads were mapped onto the BAL062 reference using smalt v0.7.4 and the resulting mapped reads were used for differential expression analysis, which was performed using DESeq (v. 1.8.2) [26]. Read counts from the col^R^ cultures, grown in the presence (64 or 128 mg l^−1^) or absence of colistin, were compared to the colistin-naïve initial culture to determine the genes with altered expression during col^R^. In addition, the initial colistin-naïve culture was compared to the BAL062 reference to remove genes with differential expression in response to ageing on solid media. Genes with a log_2_ fold-change (log_2_FC) of >1.5 (increased expression) or a log_2_FC ≤1.5 (decreased expression) and a *q* value <0.05 were considered in this analysis.

### Transposon mutant library generation and sequencing

The transposon mutant library in WT BAL062 was generated using an EZ:Tn*5* transposon containing a kanamycin-resistance cassette (Epicentre Biotechnologies), as described previously [27]. The colony number was estimated and cells batch pooled as described in [28] to yield a total of 600 000 cells in the library. For the experiment, approximately 1×10^9^ cells were inoculated into 10 ml MH broth containing 0.05 mg colistin sulphate l^−1^ (1/10th MIC; Sigma-Aldrich) and incubated overnight in a shaking incubator at 37 °C. The control did not contain colistin and experimental conditions were assessed in duplicate. The culture was serially passaged, taking 100 µl and inoculating 10 ml fresh broth with the same amount of colistin or none for the control. After an overnight incubation, DNA was extracted from 2 ml culture using the Wizard genomic DNA purification protocol (Promega) and sequenced as previously described [29].

### Analysis of transposon-directed insertion site sequencing (TraDIS) data

TraDIS sequence analysis was performed as previously described [29, 30]. Significant differences in mutant frequencies between the colistin-exposed library and the control were determined by using the edgeR package [31]. Only genes with a fold-change (log_2_FC) of >2 were considered and a corrected *P* value (*q* value, Benjamini Hochberg, of <0.05). Table S4 shows the insertion frequency results of the base library used in all challenge experiments.

### Recombination testing

We reconstructed a phylogenetic tree (midpoint rooted) of the col^R^ organisms and a subset of isolates from VAP (*n*=50) and asymptomatic carriage (*n*=16) from a previously reported study [16] using RAxML v7.8.6 with the GTRGAMMA model [32] and putative recombination blocks were predicted using gubbins, as previously described [33]. Results were viewed using Phandango (http://jameshadfield.github.io/phandango/)[34].

## Results and Discussion

### Intrinsic mediators of colistin resistance in *A. baumannii*

We used a TraDIS screen to investigate the intrinsic mediators of col^R^ by exposing a col^S^ *A. baumannii* transposon library to a sub-inhibitory concentration of colistin. High-throughput transposon mutagenesis methods, such as TraDIS, have been commonly used to assay for essential genes and genes required for survival in a given experimental condition (reviewed in [35]). We constructed a Tn*5* library in a GC2 isolate, BAL062, from a patient with VAP. The library consisted of >115 000 unique mutants (roughly 1 insertion every 35 bp). We sequenced the base library and identified 445 essential genes (Table S4) that had an insertion index ≤0.0047, accounting for ~12 % of the genome. This base library was used in all subsequent TraDIS experiments in the presence or absence (control) of colistin sulphate (0.5 mg l^−1^). Candidate genes required for colistin tolerance (where mutants are lost under the experimental conditions) or those whose loss is beneficial in the presence of colistin (where mutants expand under the experimental conditions) were determined as previously described [28, 29]. We identified 22 candidate genetic loci that were required for colistin tolerance (Table 1). The identified loci included genes that were directly involved in LOS synthesis (*lpxO* and *lpsC*) and peptidoglycan synthesis (*mcrB* and *galE*), and genes involved in transcription (*sigX*) and translation (*rplN*, *rpmA* and *rpsO*). We also identified a glycosyl transferase, *mfpsA* (similar to *lpsB*), which has been previously identified in transposon mutagenesis studies as being important for LOS synthesis [36]. Notably, *sigX* in *Bacillus subtilis* has been shown to be involved in modifying the cell envelope and conferring resistance to cationic antimicrobial peptides [37]. Additionally, a recent study using TraDIS to investigate candidate genes involved in colistin resistance identified multiple genes involved in membrane biogenesis and cellular integrity as important for colistin tolerance [38].

**Table 1.** *A. baumannii* genes involved in colistin tolerance identified by TraDIS

Locus tag	Gene name	Predicted function	log_2_FC	*q* value
Candidate genes involved in colistin tolerance				
BAL062_00718		Hypothetical protein	−4.46	5.99*e*−08
BAL062_00584		Glycosyltransferase, uncharacterized protein conserved in bacteria	−4.36	6.56*e*−12
BAL062_00901	*sigX*	RNA polymerase factor sigma-70	−4.31	1.51*e*−07
BAL062_00589		Glycosyltransferase involved in LOS biosynthesis	−3.82	1.17*e*−22
BAL062_03855	*tuaD_1*	Udg/UDP-glucose 6-dehydrogenase	−3.81	2.87*e*−04
BAL062_03869	*tuaD_2*	UDP-glucose/GDP-mannose dehydrogenase	−3.81	9.04*e*−10
BAL062_03850	*manB*	Phosphomannomutase	−3.78	4.25*e*−06
BAL062_00588		Hypothetical protein	−3.63	5.50*e*−12
BAL062_00587		UDP-d-galactose: (glucosyl)lip opolysaccharide-1,6-d-galactosyltransferase, glycosyl transferases group 1	−3.59	1.46*e*−21
BAL062_00586	*lpsC*	Lipooligosaccharide core biosynthesis glycosyl transferase, glycosyl transferase family 2	−3.59	2.07*e*−21
BAL062_03854	*pgi*	Glucose-6-phosphate isomerase	−3.52	6.12*e*−08
BAL062_03856	*galU*	UTP-glucose-1-phosphate uridylyltransferase	−3.37	3.83*e*−02
BAL062_03481	*mfpsA*	Lipopolysaccharide 1,2-*N*-acetylglucosaminetransferase, glycosyl transferases group 1	−3.34	5.86*e*−05
BAL062_01632	*rseP*	Putative membrane-associated Zn-dependent proteases 1	−3.29	8.93*e*−07
BAL062_03605	*lpxO*	β-Hydroxylase, aspartyl/asparaginyl β-hydroxylase	−3.25	3.70*e*−06
BAL062_03853	*galE_2*	UDP-glucose 4-epimerase	−3.11	3.19*e*−29
BAL062_00384	*mlaC*	Toluene tolerance protein (Ttg2D)/*mlaC*	−2.79	2.93*e*−08
BAL062_01261	*mrcB*	Murein polymerase, penicillin-binding protein 1B	−2.77	4.92*e*−12
BAL062_00383	*ttg2C*	Toluene tolerance efflux transporter (ABC superfamily)/*mlaD*	−2.66	2.37*e*−10
BAL062_00585	*icaB*	Putative polysaccharide deacetylase, poly-β-1,6-*N*-acetyl-d-glucosamine *N*-deacetylase precursor,	−2.47	1.79*e*−02
BAL062_00381	*ttg2A*	Toluene tolerance efflux transporter (ABC superfamily)/*mlaF*	−2.45	3.32*e*−13
Candidate genes contributing to colistin sensitivity				
BAL062_03783	*guaA_3*	GMP synthase, GMP synthase [glutamine-hydrolysing]	5.86	6.64*e*−02
BAL062_03861	*lst*	Putative polysaccharide biosynthesis protein, glycosyltransferase family 52	5.86	6.64*e*−02
BAL062_02787		Hypothetical protein	4.23	6.01*e*−03
BAL062_03154	*ligA*	DNA ligase	3.83	4.02*e*−02
BAL062_00203		Putative NAD/FAD-binding protein	2.65	3.33*e*−05
BAL062_03033		Hypothetical protein	2.63	2.58*e*−02
BAL062_01328		TetR family transcriptional regulator, transcriptional regulator BetI, transcriptional repressor	2.31	6.68*e*−02
BAL062_01331		Protein CsuB, uncharacterized secreted protein, spore coat protein U domain	2.22	4.81*e*−02
BAL062_01669		Glycolate/propanediol utilization protein, hypothetical protein, domain of unknown function (DUF336)	2.17	9.73*e*−02
BAL062_01330		Protein CsuA, uncharacterized secreted protein	2.13	5.27*e*−02
BAL062_01329		Protein CsuA/B, uncharacterized secreted protein, spore coat protein U domain	2.03	2.57*e*−02
BAL062_01333	*caf1A*	Protein CsuD, F1 capsule-anchoring protein precursor, fimbrial outer-membrane usher protein PefC, fimbrial usher protein	1.93	1.32*e*−02
BAL062_01332		P pilus assembly protein, chaperone PapD, putative chaperone protein EcpD, Gram-negative pili assembly chaperone, N-terminal domain	1.88	8.15*e*−02

Candidate genes involved in maintaining cell-surface lipid symmetry [39] were also amongst those that we identified to be required for tolerance to colistin, including *ttg2D*/*mlaC*, *ttg2A/mlaF* and *ttg2C/mlaD* (Table 1). Other genes within the *mla* locus were just below the stringent log_2_FC ≤2 (*q* value <0.05) cut-off for genes required for tolerance (Table S5). Previous studies have found that the *mlaBCD* genes are upregulated in col^R^ *A. baumannii* strains lacking LOS [40] and deletion of any component of the Mla pathway results in outer-membrane (OM) instability [39]. These data highlight the critical role of maintaining the lipid component and stability of the OM in colistin-susceptible organisms in *A. baumannii*.

Candidate genes that were thought to contribute to colistin susceptibility (i.e. the disruption of genes beneficial for survival in the presence of colistin) included putative pilus assembly genes (BAL062_01329, *caf1A* and BAL062_01332) and a gene encoding a TetR family protein (BAL062_01328) with 99 % DNA sequence similarity to *adeN*. Previous studies have reported a decrease in the expression of OM structures, such as pili, in response to disruption or damage of the OM that may act to limit membrane spanning surface structures to maintain cellular integrity [40]. AdeN is member of the TetR transcriptional repressor family and has been shown to regulate the expression of *adeIJK* efflux proteins [41], suggesting the involvement of efflux for survival in the presence of sub-inhibitory concentrations of colistin.

### ISAbA1-mediated *mlaA*, *lpx* and *baeSR* gene disruption confers resistance to colistin

To determine the genetic changes in *in vitro* generated col^R^ organisms, two independent biological replicate cultures (C1 and C2) of *A. baumannii* BAL062 that achieved an MIC of >64 mg l^−1^ (midpoint)/ ≥128 mg l^−1^ (endpoint) were subjected to WGS (Table 2). We detected multiple ISAbA1-mediated disruptions in the *lpxC* gene in the induced col^R^ organisms (C1 and C2) grown in media supplemented with 64 and 128 mg colistin l^−1^ (Table 2). The *lpxC* gene is almost ubiquitous across Gram-negative bacteria and is an essential component of the lipid-A biosynthesis pathway. Mutations in the *lpx* genes have previously been shown to confer resistance to colistin in *A. baumannii* [11–13]. We identified only two mutations in yet uncharacterized genes, −404 upstream of a putative OMP gene, BAL062_00181 and BAL062_01694 (Q212*); however, the effect of these mutations in col^R^ or as compensatory mutations requires further investigation.

**Table 2.** ISAba1-mediated disruptions in colistin-resistant BAL062-derived

Culture	Passage day	Colistin concn (mg l^−1^)	Further growth with colistin†	Further growth without colistin†
C1	1	0	–	None
	2	32	None	None
	5	64	*lpxC* (572), *mlaA* (894)	*lpxC* (572), *lpxD* (828), *baeR* (159)
	6	128	*lpxC* (572), *mlaA* (894)	*lpxC* (572), *mlaA* (894), −404 upstream of OMP BAL062_00181
C2	2	64	*lpxC* (572), *baeS* (1033), BAL062_01694 (Q212*)	None
	5	128	lpxC (523, 270), *mlaA* (561), BAL062_01694 (Q212*)	*lpxC* (523, 270), BAL062_01694 (Q212*)

†Gene names indicate ISAba1-disrupted genes and the amino acid positions are given in brackets. Q212* is the nonsense mutation at position 212 in BAL062_01694.

We additionally observed the disruption of *mlaA* (a transmembrane protein) by ISAba1 in both the C1 and C2 col^R^ (≥128 mg l^−1^) cultures (Table 2). MlaA is an OM lipoprotein of unknown function that has been previously implicated in col^R^ [39]. The disruption of *mlaA* by ISAba1 suggests this protein contributes toward colistin tolerance. Mutations in this gene have also been observed previously in col^R^ *A. baumannii* [17]. However, this finding contradicts the results observed using TraDIS, where *mlaA* inactivation resulted in increased susceptibility. We hypothesize that maintaining cellular integrity is crucial for survival at low sub-inhibitory concentrations (as in the TraDIS experiments) and may be present in a mixed population (Fig. S1); however, once resistance is achieved (directed evolution experiments), the disruption of LOS synthesis, mediated by mutations in *lpx*, becomes the primary mechanism of resistance and *mlaA* becomes less critical as colistin can no longer bind to the negatively charged LOS polymer due to charge interactions. We hypothesize that this dynamic shift in resistance mechanisms at the different concentrations of colistin could be a novel col^R^ mechanism employed by *A. baumannii* associated with tolerance to colistin until full resistance is achieved.

**Fig. 1.**
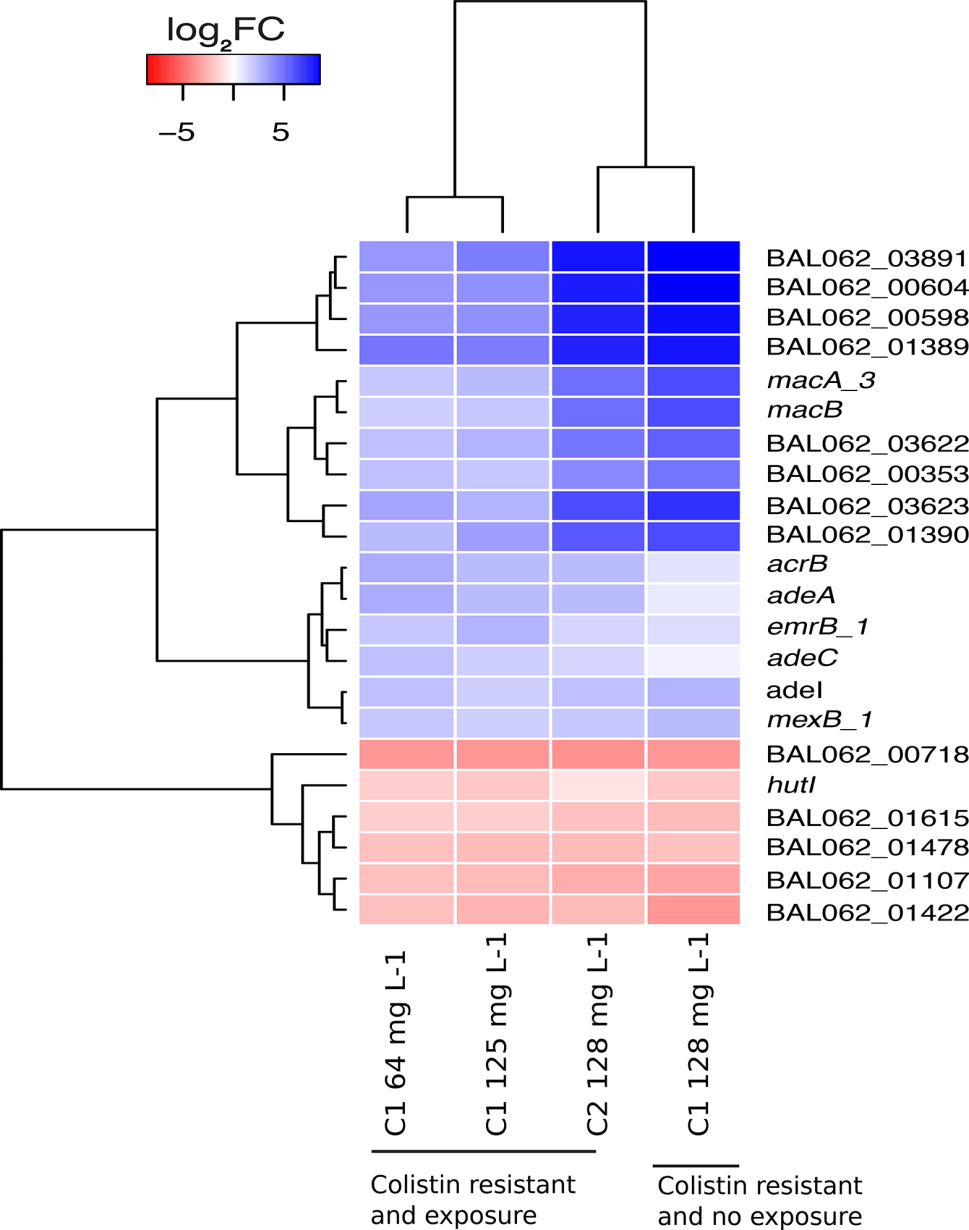
Transcriptional changes in *in vitro* generated colistin-resistant *A. baumannii.* Differentially expressed genes common to all colistin-resistant cultures (C1 and C2) grown in the presence of colistin (64 or 128 mg l^−1^) or without selection compared to the day 0 BAL062 culture (WT). The heat map, obtained using values from DESeq2 analysis, excludes genes that were differentially expressed in the passaged BAL062 culture that was not exposed to any colistin. Blue colours indicate genes with higher expression relative to the comparator, red colours indicate lower expression.

The C1 and C2 midpoint cultures grown in the presence of 64 mg colistin sulphate l^−1^ additionally had ISAba1 disruptions in *baeS* (C2) or *baeR* (C1). These loci constitute part of a two-component system (TCS) involved in bacterial stress response and increased expression of multidrug efflux proteins [42–44]. We subjected the C1 culture (*baeR* disrupted) to RNAseq to investigate any changes in efflux. Surprisingly, we found a ~3- and ~5-fold increase in expression in *macAB* and *adeABC e*fflux systems, respectively, in the presence of 64 mg colistin l^−1^ (Fig. 1, Table S6). This is an unusual observation, and we can only hypothesize that other TCSs not yet characterized may be responsible for this observation. Another unexplained observation was the lack of genotypic changes that could account for the C1 culture able to grow in 32 mg colistin l^−1^ (Table 2). This lack of fixation of a mutant population has previously been reported in a heteroresistant *Klebsiella pneumoniae* [45]. In a recent study, Band and colleagues [46] described a loss of col^R^ of an otherwise resistant population upon removal of selection, further demonstrating the heteroresistant nature of colistin exposure to bacterial populations. We postulate that this phenomenon may also occur in *A. baumannii*, where the SNP changes would be present in a subpopulation. However, we would need to carry out further work to confirm this and explain the genetic basis of the would-be out-competition of these mutant variants by WT variants.

### Mutation in a zinc peptidase may provide an alternative colistin-resistance mechanism

We further observed a nonsense mutation (Q212) in a zinc peptidase (BAL062_01694) in one of the passage cultures (C2) when grown in 64 and 128 mg colistin l^−1^. Zinc peptidases catalyse the cleavage of peptide bonds in a metal-dependent manner [47]. The nonsense mutation in col^R^ organisms occurred upstream of the active site (E270), and likely rendered this enzyme inactive. Zinc metalloproteases have been linked in antimicrobial peptide resistance in *Burkholderia* spp. [48]. This mutation has previously been identified in a col^R^ BAL062 strain and is thought to be involved in OM processing [17].

### Mutations in the *pmr* locus confer colistin resistance in clinical isolates of *A. baumannii*

We investigated mechanisms of col^R^ in three clinical *A. baumannii* strains. These organisms were isolated in late 2012 and early 2013 from patients treated with empirical low dose colistin (2.3 mg^−1^ kg^−1^ per day). After repeated treatment failure, clinical specimens were taken and the isolates were subjected to WGS after they were found to be resistant to both colistin and meropenem. These three clinical isolates, BAL505, BAL543 and BAL719, had MIC values of 24, 16 and 64 mg colistin l^−1^, respectively. WGS indicated that all three isolates belonged to GC2, but harboured a large number of non-synonymous SNPs in *pmrB* relative to the col^S^ GC2 1652–2 reference genome.

**Fig. 2.**
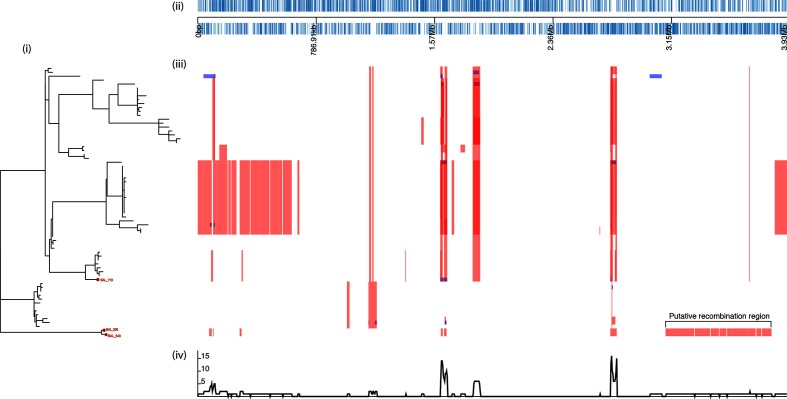
Inferred recombination in clinical isolates of colistin-resistant *A. baumannii.* These data were visualized using Phandango (https://github.com/jameshadfield/phandango/). (a) A maximum-likelihood tree of the clinical colistin-resistant organisms (BAL505, BAL543 and BAL719) and colistin-sensitive *A. baumannii* GC strains [42]. (b) Schematic representation of the reference *A. baumannii* genome. (c) Recombination regions are indicated as coloured blocks relative to the taxa involved, red blocks indicate predicted recombination that occurred on an internal branch, blue blocks are only present in one isolate (I) and the genomic region affected (II). (d) The line graph summarizes the data in (c).

On further investigation, we observed that the genomes of two of the col^R^ isolates (BAL505 and BAL543) exhibited evidence of substantial recombination with other co-circulating *A. baumannii* of approximately 700 kb in length with a total of 14 296 SNPs (Fig. 2)(Table S7). Recombination events are frequently reported in the capsule and outer core regions in MDR *A. baumannii*, and are thought to be an important source of diversification [49–51]. The putative recombinogenic region harboured the *pmr* locus, which explained the high number of SNPs. We conducted a blastn and blastp search to identify mutations that may confer a col^R^ phenotype. We found BAL505 harboured a H266Y mutation in the histidine kinase domain of *pmrB*, whilst BAL543 and BAL719 harboured mutations at positions L94W and P170L (Table 3). The P170L mutation has previously been reported in a polymyxin-resistant *A. baumannii* clinical isolate [8], the other two mutations have not been observed previously in col^R^. Mutations in the *pmrAB* locus have previously been shown to confer col^R^ in *A. baumannii* [7, 14, 52, 53].

**Table 3.** Summary of amino acid changes identified in colistin-resistant clinical *A. baumannii* isolates

Organism	Amino acid changes in PmrB	MIC (mg l^−1^)
BAL505	H266Y	24
BAL543	L94W	16
BAL719	P170L	64

### Transcriptional analysis of *in vitro* generated col^R^ strains

We compared genes that were differentially expressed between the two independent col^R^ organisms in the presence and absence of colistin, midpoint and endpoint for C1 and endpoint for C2 (Tables S1 and S8). To rule out changes in expression as a result of passaging, we compared aged colonies to an independent culture maintained over the same period without antimicrobial selection.

#### Genes with decreased expression

Col^R^ cultures (C1 and C2) grown in either the presence (64 and 128 mg l^−1^) or absence of colistin exhibited a decreased expression of genes involved in metabolic processes such as histidine utilization, i.e. *hutUHI*, fatty acid catabolism and the CoA thioester intermediates (e.g. *paa*, *echA8* and *mgh*) (Table S6). The *hut* genes are involved in the formation of formiminoglutamate and is an essential amino acid in protein synthesis [54, 55], whereas fatty acid catabolism is involved in the breakdown of LOS, the primary target for colistin [6]. We expected that the decreased expression of these genes reduced the destructive impact of colistin exposure reinforcing the cell membrane and maintaining cellular integrity.

#### Genes with increased expression

Differentially expressed genes between the different col^R^ cultures (C1 and C2) grown in the presence of colistin, at 64 or 128 mg l^−1^, or in the absence of colistin were also compared. Genes common to all four conditions (col^R^ +/− colistin) and not in aged colonies (control) were considered. A 2–11-fold (log_2_FC 1.5–5.8) increase in expression was observed in genes encoding the efflux proteins *adeI*, *adeC*, *emrB*, *mexB/adeJ* and *macAB* relative to the parent strain when not exposed or grown in colistin (Fig. 1, Table S6). Previous studies have also seen an increase in expression of efflux proteins *adeIJK* and *macAB-tolC* in a *lpx-*deficient *A. baumannii* strain in the presence of colistin [40]. In in our study, *adek* (BAL062_00757) and *tolC* (BAL062_03655) did not meet the minimum threshold (Table S8).

Notably, a transcriptional regulator *nemR*, which is upstream of *macAB* and part of the TetR family, exhibited a 3–6-fold decrease in expression in the col^R^ cultures, indicating that this efflux system may be under the control of *nemR*, a common feature of the TetR repressors [56]. Efflux is a collective mechanism employed by bacteria to confer resistance to multiple drugs including colistin in *A. baumannii* [57–59]. We hypothesize that along with the involvement of *mla* in subinhibitory concentrations in the TraDIS experiment, initial response to colistin is primarily by efflux until lipid-A synthesis is disrupted due to the ISAba1-mediated disruption of *lpxC,* where both resistance mechanisms may function together. Further experiments will be needed to confirm this.

Other genes with increased expression included those encoding putative signal peptides BAL062_00353, BAL062_00598 and BAL062_03891, an acid shock protein (BAL062_00604), two 17 kDa surface antigens (BAL062_01389 and BAL062_01390), and a lipoprotein (BAL062_03623). These genes had a 4–17-fold increase in expression. Future work is required to elucidate the function of these genes and their role in colistin resistance/tolerance.

### Conclusion

Our analysis showed that multiple mechanisms are associated with intrinsic and acquired mechanisms of col^R^ in *A. baumannii*. Genes involved in the maintenance of cellular integrity appear to be crucial in permitting bacteria to survive in sub-inhibitory concentrations of colistin, in addition to genes involved in peptidoglycanand LOS synthesis. The col^R^ mechanisms outlined above were mainly associated with ISAba1 or mutational changes in genes critical for cell wall synthesis or genes controlling their expression. This included the disruption of *baeR,* a TCS, resulting in the increased expression of efflux pumps, such as MacAB and AdeIJK. Drug efflux is an important resistance mechanism in bacteria, and has recently been identified in mediating col^R^ in *A. baumannii* [60]. A recent study using an efflux pump inhibitor found that carbonyl cyanide m-chlorophenyl hydrazine (CCCP) reversed and/or suppressed col^R^ in *A. baumannii* [61]. Although this chemical compound is not suitable for clinical use, our work highlights the crucial role of MDR efflux pumps in acquired col^R^, which could be useful targets for future therapies to be used in combination with colistin to maintain the efficacy of this crucial last-line therapy. Our results have described the complex yet linked nature of colistin tolerance and col^R^, highlighting the interaction of a multitude of effectors and stress-related genes required to generate this phenotype.

## Data Bibliography

1. Sequence data (WGS and RNAseq) from this study were deposited in the European Nucleotide Archive and the accession numbers may be be found in Table S2.
2. The complete genome sequence for *baumannii*GC2 1652-2 was obtained from Park JY, Kim S, Kim S-M, Cha SH, Lim S-K, *et al.* Complete genome sequence of multidrug resistant *Acinetobacter baumannii* strain 1656-2, which forms sturdy biofilm. *J Bacteriol* 2011;193:6393–4.
