# Supplementary material for: Clinical and laboratory-induced colistin-resistance mechanisms in Acinetobacter baumannii
Source: Microb Genom. 2019 Feb 5;5(2):e000246. doi: 10.1099/mgen.0.000246 (PMC6421349; doi:10.1099/mgen.0.000246)
Supplement: Supplementary File 1 [file mgen-5-246-s001.pdf]

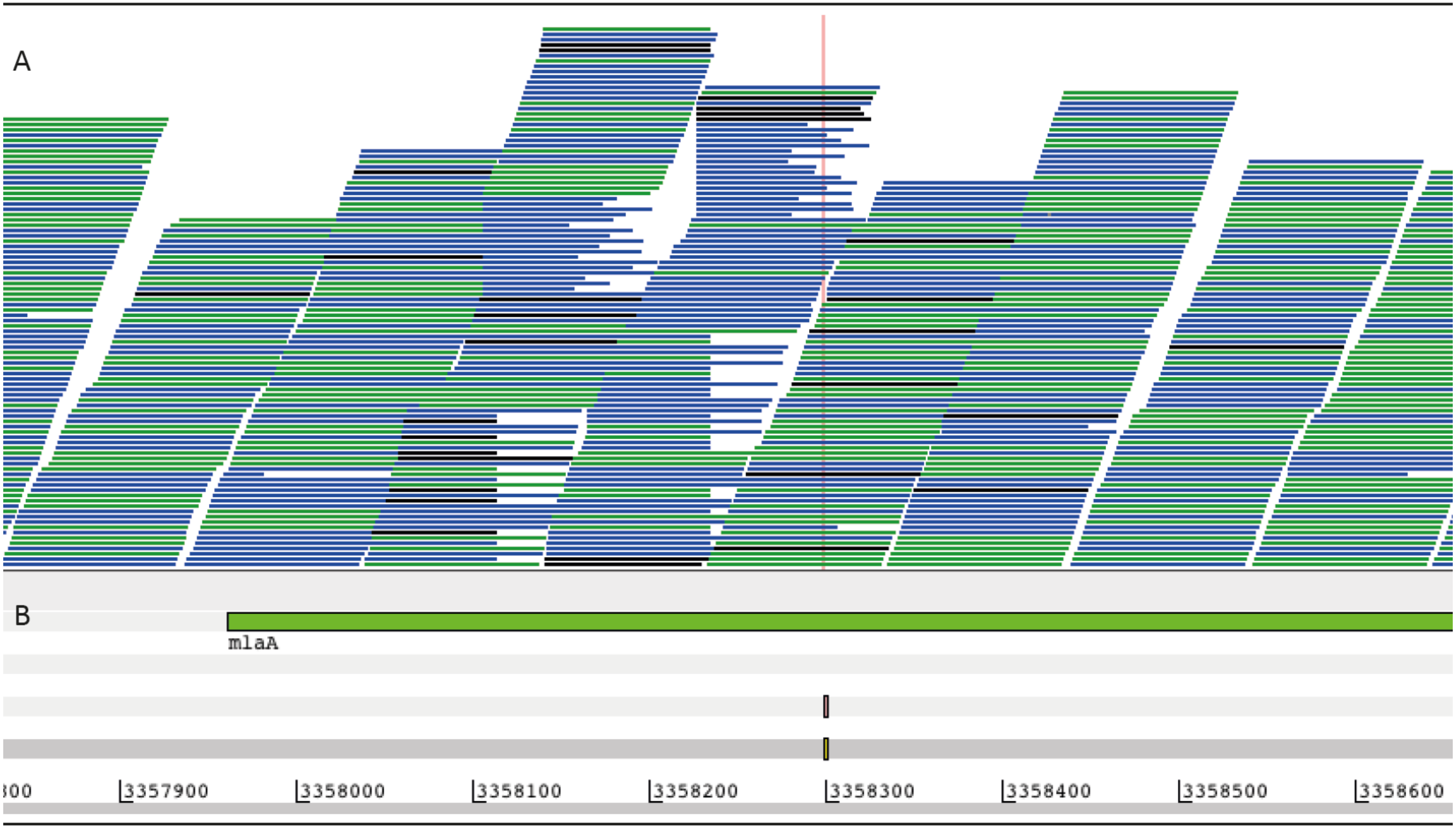

Figure S1: Artemis screen shot of the region of IS*Aba1* disruption in *mlaA* of Col^{R} culture 2 grown in 128mg/L colistin**.**
The reads were mapped onto the BAL062 reference genome (panel A). Highlighted is the insertion point of IS*Aba1* that occurs between 3358299 - 3358301 bp in *mlaA* (panel B). Illustrated are reads mapping across the insertion point in *mlaA* indicating a population of wild type cells. The mixed (minority) mutant population may result in growth of the wild type cells being favored in the absence of colistin.
